# High-Precision Detection of Cellular Drug Response Based on SERS Spectrum and Multivariate Statistical Analysis

**DOI:** 10.3390/bios13020241

**Published:** 2023-02-08

**Authors:** Fengfang Wu, Zhiwei Wu, Xiaoyan Wang, Yunliang Liu, Qing Ye

**Affiliations:** 1 Shengli Clinical Medical College of Fujian Medical University, Fuzhou 350001, China; 2 Department of Otolaryngology, Head and Neck Surgery, Quanzhou First Hospital Affiliated to Fujian Medical University, Quanzhou 362000, China; 3 Department of Otolaryngology, Head and Neck Surgery, Fujian Provincial Hospital, Fuzhou 350001, China

**Keywords:** nasopharyngeal carcinoma, secreted protein, cisplatin, surface-enhanced Raman scattering (SERS)

## Abstract

The rapid development of personalized medicine places high demands on the control of drug dose and cellular drug response to provide patients with better curative effects and low side effects. To solve the problem of low detection accuracies of the cell-counting kit-8 (CCK8) method, a detection method based on surface-enhanced Raman spectroscopy (SERS) of cell-secreted proteins was adopted to evaluate the concentration of the anticancer drug cisplatin and the cellular drug response of nasopharyngeal carcinoma. CNE1 and NP69 cell lines were used to evaluate cisplatin response. The results showed that the combination of the SERS spectrum with principal component analysis–linear discriminant analysis could detect the difference in the response of cisplatin with a concentration difference of 1 μg/mL, which considerably exceeded that of CCK8. In addition, the SERS spectral peak intensity of the cell-secreted proteins strongly correlated with the cisplatin concentration. Furthermore, the mass spectrum of the secreted proteins of the nasopharyngeal carcinoma cells was analyzed to verify the results obtained using the SERS spectrum. The results demonstrated that SERS of secreted proteins has great potential for high-precision detection of chemotherapeutic drug response.

## 1. Introduction

Nasopharyngeal carcinoma (NPC), which occurs mostly in Southeast Asia and Southern China, is malignant and fatal [1]. According to statistics from the World Health Organization, approximately 70% of the world’s patients with newly developed nasopharyngeal cancer in 2018 belonged to these regions [2,3]. Chemotherapy and radiation therapy are currently the first-line treatment options for nasopharyngeal carcinoma [4,5,6]. A large number of patients will achieve clinical remission after therapy. However, approximately 30% of patients will suffer from locoregional recurrence or distant metastasis. Metronomic low-dose chemotherapy was considered to be a potential strategy for the treatment of patients with advanced nasopharyngeal carcinoma. The results of a parallel-group, randomized, controlled, phase 3 trial, which were published by Jun Ma et al. in 2021 [7], showed that the application of Metronomic capecitabine after definitive chemoradiotherapy could dramatically improve failure-free survival time and quality of life in patients with high-risk locoregionally advanced nasopharyngeal carcinoma. However, the mechanism by which metronomic low-dose chemotherapy inhibited tumor cells and modulated cell microenvironment was still unknown.

Previous studies indicated that secreted proteins may be potential targets for the research of metronomic low-dose chemotherapy. Cell-secreted proteins are important biological macro-molecules which include cytokines, immune regulators, hormones, and other biologically active molecules that participate in many important life processes, for example, modulation of cell microenvironment, cell proliferation and differentiation, immune response, lipid metabolism, and cell signaling [8,9,10,11,12]. Cell-secreted proteins are closely associated to the angiogenesis, differentiation, invasion, and metastasis of malignant tumors. In 2021, Kocaturk et al. [12] found that the composition of nasal secreted proteins changed once treated with lipopolysaccharide, reflecting different mechanisms, such as oxidative stress, activation of immune responses, ubiquitin pathway, and SUMOylation. Added choline could change the response of nasal secreted proteins to lipopolysaccharide. Traditionally, analysis methods of secreted proteins needed long operation times, complicated operation steps, or higher costs, including enzyme-linked immunosorbent assays (ELISAs), Western blot, mass spectrometry, etc.

Raman spectroscopy (RS), especially surface-enhanced RS (SERS), which is also known as “fingerprint cryptography,” can sensitively reflect subtle changes in internal molecular components and structures of substances through changes in the Raman spectrum peak. SERS is non-invasive, direct, accurate, and rapid and has been widely studied in the biomedical field in recent years. In this regard, RS has made great progress in the applications of drug screening, drug efficacy evaluation, toxicity evaluation, and other fields [13,14,15,16]. In 2020, Qiu et al. [15] evaluated the therapeutic effect of chemotherapy drugs for common nasopharyngeal cancer on CNE2 and C666-1 cells using a laser optical tweezer RS measurement system. The authors reported that laser optical tweezers RS has high accuracy, sensitivity, and specificity and can effectively identify and distinguish the types of nasopharyngeal cancer cells [15]. In 2021, Wang et al. [16] used deuterium isotope labeling to conduct Raman detection in a single cell, which can not only accurately determine the effect of drugs but also shorten the test period from 72–144 h to 48 h.

Furthermore, SERS was also used to analyze serum protein [17] and saliva protein [18]. These studies show that laser RS has good application potential in metronomic low-dose chemotherapy.

At present, the literature on accurately evaluating the cellular response of low-dose cisplatin to nasopharyngeal carcinoma cells, especially with regard to possible differences in amino acid metabolism, using secreted protein-SERS is absent. We believe that this study can provide some therapeutic inspiration to doctors.

## 2. Experimental Preparation

### 2.1. Cell Culture

In this study, a highly differentiated human nasopharyngeal carcinoma cell line (CNE1) and normal nasopharyngeal human epithelial cell line (NP69) (purchased from Xiang Ya Central Experiment Laboratory, Changsha, China) were used as experimental and control groups, respectively. Cisplatin (Cat. No. A8321) was purchased from APExBIO Company, Houston, TX, USA.

A total of 10% fetal bovine serum (FBS) and 100 U/mL of penicillin and streptomycin were added to the culture cell line in the dish containing RPMI-1640 medium. The dish was placed in an incubator and humidified (5% CO_2_ at 37 °C). The medium was changed every other day. When the cell density reached 80%, the cells were passaged.

### 2.2. Assessment of Cell Viability

The CCK8 method, which is widely applied in cytotoxicity tests, was adopted to assess cell viability [19]. When NP69 and CNE1 cells grew at the logarithmic stage, the cells were first harvested, centrifuged at 1000× *g* rpm/min for 5 min, washed with 1 × phosphate-buffered saline (PBS) three times, and subsequently resuspended as a single cell suspension in 2 mL RPMI-1640. The cell suspension was seeded into a 96-well plate with 1 × 10^4^ cells per well (100 μL). When the cells were grown as a single layer in the plate bottom, the medium was discarded, and the cells were washed with 1 × PBS three times to remove any residual medium. Subsequently, a 100 μL RPMI-1640 medium with different concentrations of cisplatin was added to every well.

To study the cellular drug response of cisplatin, a control group and three experimental groups were established. The cisplatin concentrations used in the experiment ranged from 0 to 5 μg/mL. Each group contained five samples. Cell-secreted proteins were extracted from the culture supernatants after 24 h of cultivation. Next, the cell viability was assessed using the CCK8 method (Dalian Meilun, Dalian, China).

### 2.3. Preparation of Secreted Proteins

The cell suspension was inoculated into a six-well plate with a density of 3.75 × 10^5^ per well (total volume: 150 μL). After 24 h, when the cells had grown to 80% density, the medium was removed, and the cells were washed with 1 × PBS three times to remove any residual medium. Different cisplatin concentrations in 2 mL RPMI-1640 medium were added to each well. The secreted proteins were extracted from the culture supernatants after 24 h. In this regard, the 0, 3, 4, and 5 μg/mL groups were extracted in both NP69 and CNE1 cell lines. The secreted proteins were purified using ethanol extraction [20]. Twice the amount of anhydrous ethanol was added to the cell supernatant and mixed fully. After centrifugation at 4 °C and 12,000× *g* rpm/min for 8 min, the supernatant was discarded, and the white precipitate that was attached to the bottom of the centrifuge tube could be seen. Subsequently, the precipitate was washed once with 95% ethanol by centrifugation at 4 °C and 10,000× *g* rpm/min for 10 min. The supernatant was discarded to obtain a precipitate in the form of a white lump at the bottom of the centrifuge tube. Next, the precipitate was dried at 25 °C for 1–2 h to fully volatilize the ethanol. A total of 40 µL ultrapure water was added to dissolve the dried cell-secreted proteins to obtain a uniform secreted protein solution.

### 2.4. Preparation of Silver Colloids

The silver colloids used in this experiment were synthesized using the method described in the study by Lin et al. [18]. First, a 4.5 mL sodium hydroxide solution and 5 mL 0.06 mmol/l hydroxylamine hydrochloride solution were mixed. Second, the mixture was rapidly added into a 90 mL 0.0011 mmol/l silver nitrate aqueous solution. Third, the mixture was stirred for 10 min to obtain gray silver colloids. Finally, the silver colloids were obtained by centrifuging the solution at 10,000× *g* rpm/min for 8 min, and the supernatant was subsequently discarded.

### 2.5. SERS Measurements

The secreted protein solution was mixed as uniformly as possible with the silver colloids in a 1:1 ratio using an Eppendorf pipette. The mixture was then transferred to a clean and smooth aluminum plate (Guantai Metal, Langfang, China) for detection. A confocal Raman microscope (inVia System, Renishaw plc, Gloucestershire, U.K.) was used to measure the SERS spectra in the range of 400–1800 cm^−1^. The selected wavelength of the diode laser was 785 nm. The SERS signals were collected with a 10 s integration time by a 20 × objective lens. Before the SERS measurement, the first-order peak at 520 cm^−1^ of monocrystalline silicon was used as the reference peak position for calibrating the spectral peak. The data were acquired and analyzed using WIRE 3.4 (Renishaw plc, Wotton-under-Edge, UK).

### 2.6. Mass Spectrometry

The obtained secreted proteins were identified using mass spectrometry (Q Exactive HF-X Mass Spectrometer, Thermo, Waltham, MA, USA), which confirmed that the substances we extracted were indeed proteins.

### 2.7. Statistics Method

Data were presented as mean ± standard error from at least three independent experiments. The student’s t-test was used to evaluate the significance of the difference between the two groups. The software SPSS 19.0 (SPSS, Chicago, IL, USA) and Origin 8.5 (OriginLab Corporation, Northampton, MA, USA) were used for statistical and graphic analyses. Additionally, *p* < 0.05 was defined as a statistically significant difference.

## 3. Results

### 3.1. Results of Silver Colloids

**Figure 1:**
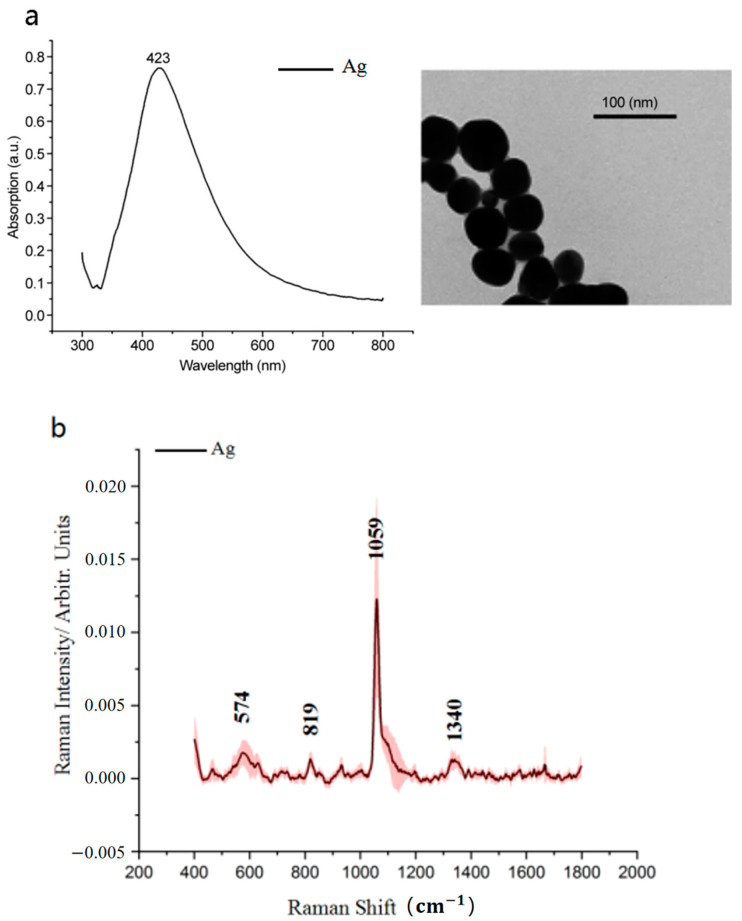
(**a**) UV−visible−NIR absorption spectrum and TEM micrograph of Ag colloids. (**b**) Mean SERS spectra of the Ag colloids.

The maximum absorption peak of the silver colloids was located at 423 nm. The average diameter of the silver colloid nanoparticles observed using transmission electron microscopy (TEM) was 38 nm (see Figure 1a). The mean SERS spectra of the Ag colloids was shown in Figure 1b. The peaks of the Ag colloids exist mainly at 574, 819, 1059, and 1340 cm^−1^.

### 3.2. Results of Cell Viability of CCK8

**Figure 2:**
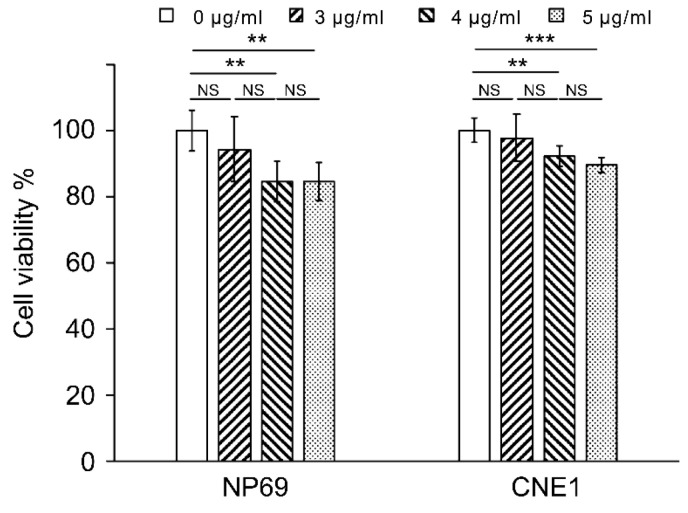
Statistical results of the cell viabilities of NP69 and CNE1 treated with cisplatin concentrations in the range of 0–5 μg/mL. NS—no statistical significance, *p* > 0.05, ** *p* < 0.05, and *** *p* < 0.01.

Figure 2 shows that the viabilities of both types of cells decrease significantly after 24 h as the drug concentrations increase. However, the cell viability of NP69 treated with 3, 4, and 5 μg/mL cisplatin is 94.23%, 84.52%, and 84.66%, respectively, and no significant differences are found between each group (*p* > 0.05). Meanwhile, the cell viability of CNE1 treated with 3, 4, and 5 μg/ mL cisplatin is 97.71%, 92.26%, and 89.58%, respectively, and no significant differences between the 3 and 4 μg/mL groups, or between the 4 and 5 μg/mL groups (*p* > 0.05), were found.

Our experiment shows that when the concentration difference is 1 µg/mL, the CCK8 method cannot sufficiently evaluate the cellular drug response. Hence, we introduced SERS technology to distinguish the cellular drug response of cisplatin on different types of cell lines.

### 3.3. Results of Mass Spectrometry

**Figure 3:**
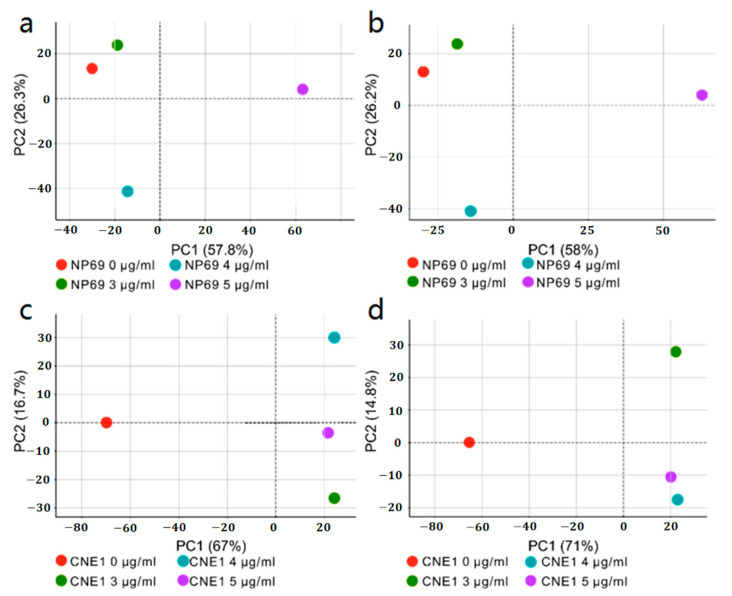
(**a**) Quantitative principal component analysis (PCA) diagram of NP69 secreted proteins. (**b**) PCA diagram of the differences among the NP69 secreted proteins. (**c**) Quantitative PCA diagram of CNE1 secreted proteins. (**d**) PCA diagram of the difference in CNE1 secretion proteins.

Table 1 lists the details. In addition, the differences between the secreted proteins of CNE1 and NP69 were analyzed, as shown in Figure 3.

### 3.4. SERS Spectral Analysis of Cell-Secreted Proteins

Raman peaks, which are also known as “molecular fingerprints”, can reflect material composition in detail. The peaks represent specific chemical bonds or some particular functional groups in the substance [21].

#### 3.4.1. SERS Spectra of CNE1 Cell-Secreted Proteins

**Figure 4:**
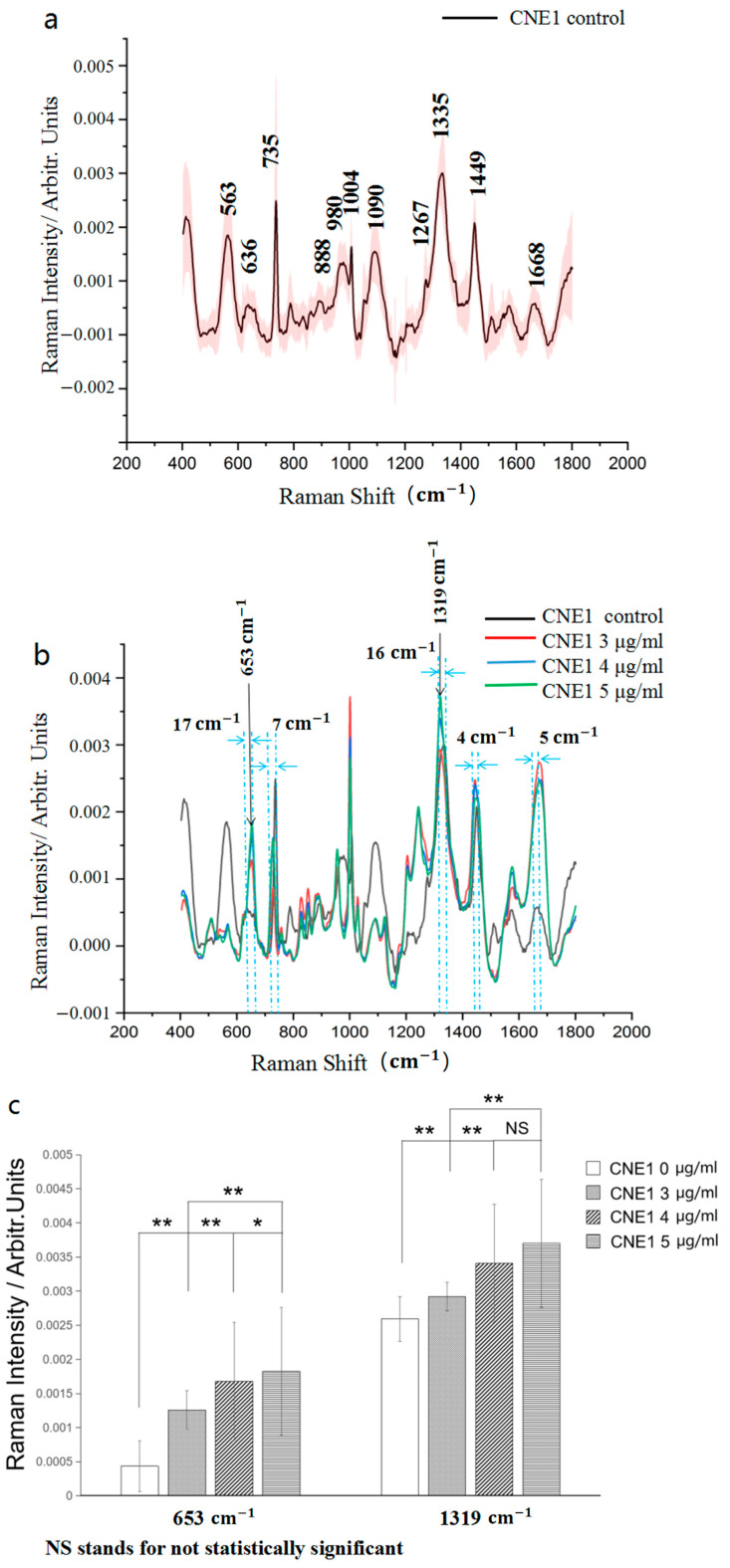
(**a**) Mean SERS spectra of the CNE1 cell-secreted proteins in the control group. (**b**) Mean SERS spectra of the CNE1 cell-secreted proteins from the control group and three experimental groups (3, 4, and 5 μg/mL) and the offset. (**c**) Histogram of the Raman peak intensities at 653 and 1319 cm^−1^ from the three experimental groups. NS stands for not statistically significant, * represents *p* < 0.05, and ** represents *p* < 0.001.

Figure 4a shows the mean SERS spectra of CNE1 cell-secreted proteins cultured using the RPIM-1640 medium for 24 h (*n* = 60). The peaks of the secreted proteins from CNE1 exist mainly at 563, 636, 735, 888, 980, 1004, 1090, 1267, 1335, 1449, and 1668 cm^−1^. Figure 4b displays the mean SERS spectra of the CNE1 cell-secreted proteins treated with different concentrations of cisplatin (*n* = 60). Figure 4c shows that the peaks increase with an increase in the cisplatin concentration at 653 and 1319 cm^−1^ (*p* values < 0.05).

According to Figure 2, although the cell viability does not differ between the 3, 4, and 5 μg/mL groups, the SERS spectra are different from each other. This indicates that SERS can identify the drug response of different concentrations of cisplatin on CNE1 cells because this method is more sensitive than the CCK8 assay.

#### 3.4.2. SERS Spectra of the NP69 Cell-Secreted Proteins

**Figure 5:**
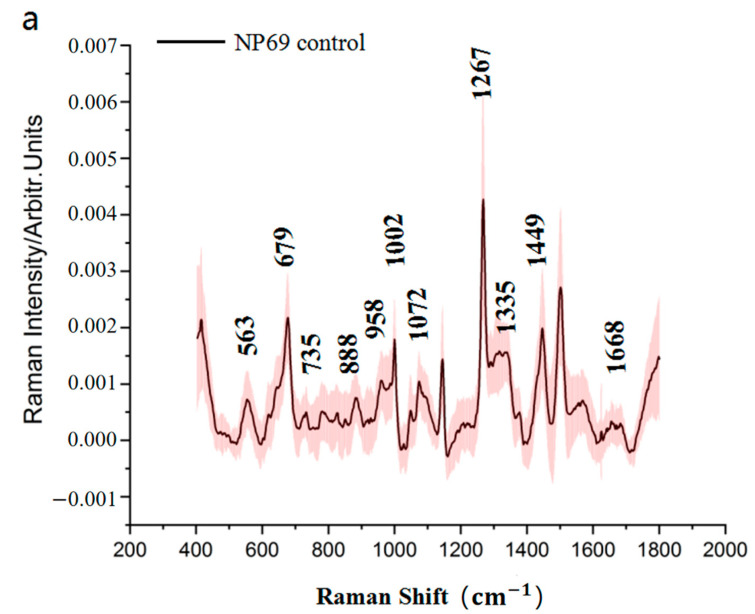

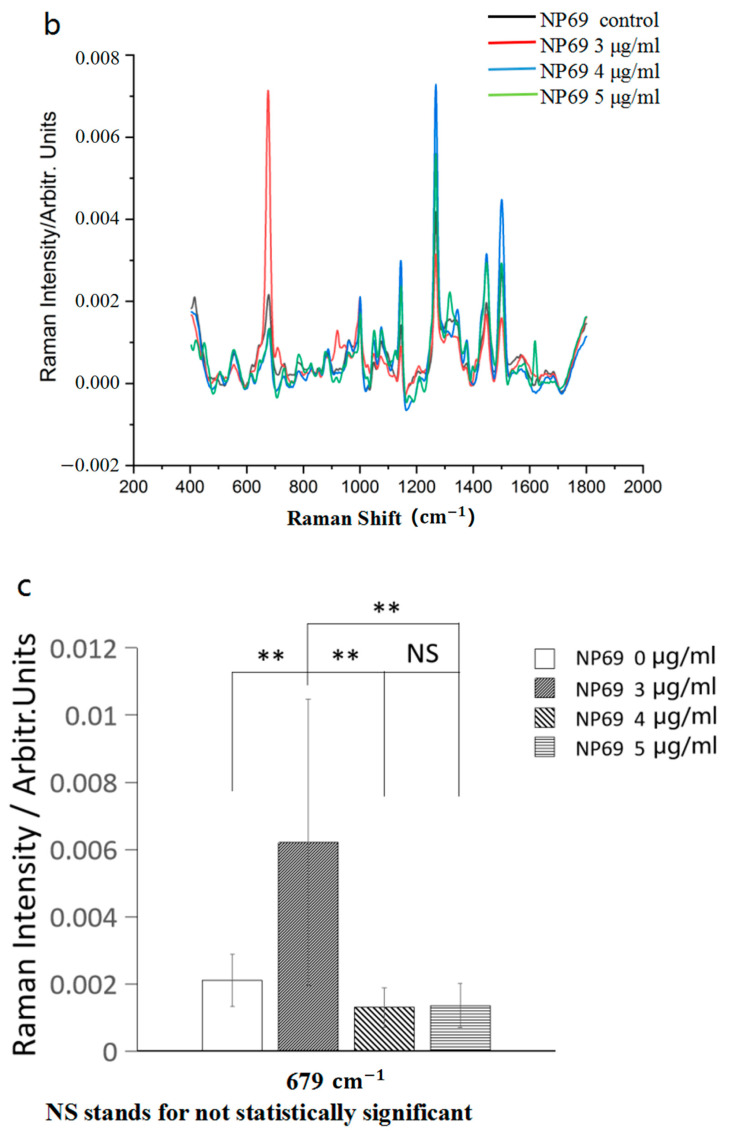
(**a**) Mean SERS spectra of the NP69 cell-secreted proteins in the control group. (**b**) Mean SERS spectra of the NP69 cell-secreted proteins from the control group and three experimental groups (3, 4, and 5 μg/mL). (**c**) Histogram of the Raman peak intensities at 679 cm^−1^ from the three experimental groups. NS stands for not statistically significant, and ** indicates *p* < 0.001.

Figure 5a shows the average SERS spectra of NP69 cell-secreted proteins cultured using the RPIM-1640 medium for 24 h (*n* = 30). The peaks of the secreted proteins from NP69 in the natural state exist mainly at 563, 679, 735, 888, 958, 1002, 1072, 1267, 1335, 1449, and 1668 cm^−1^. Figure 5b displays the average SERS spectra of the NP69 cell-secreted proteins treated with different concentrations of cisplatin (*n* = 30). Figure 5c shows that the peaks decline drastically at 679 cm^−1^.

**Figure 6:**
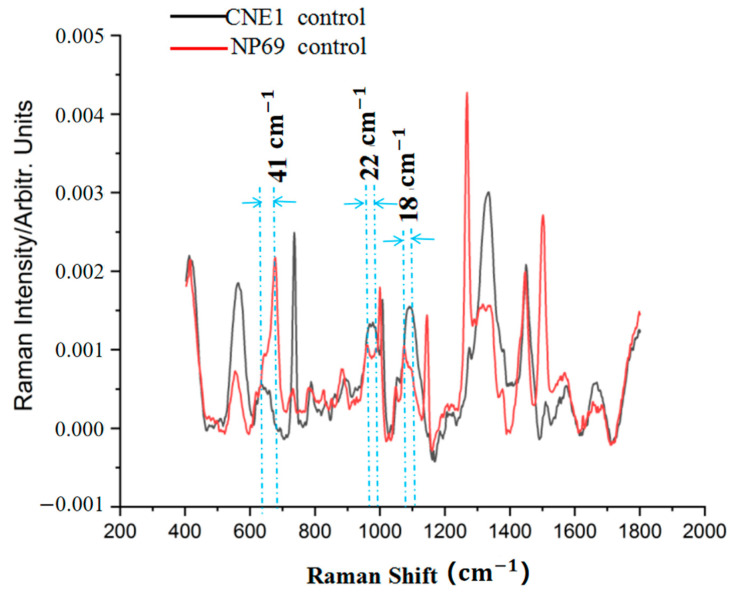
Mean SERS spectra of the CNE1 and NP69 cell-secreted proteins in the control group and the offset of Raman shift.

Furthermore, we compared the Raman spectra of CNE1 and NP69 cell-secreted proteins in the control groups and marked the offset, as shown in Figure 6.

#### 3.4.3. Tentative Peak Attribution of the SERS Spectra

Table 2 lists the tentative assignments of SERS peaks according to the literature [22,23,24,25].

### 3.5. Multivariate Statistical Analysis (PCA–LDA)

The original SERS spectra included many components, such as fluorescence background, Raman scattering, and noise signals. To eliminate meaningless components, we used the Vancouver Raman algorithm to acquire high-quality Raman spectra [18]. The adjusted SERS spectra were normalized in the range of 400–1800 cm^−1^. Furthermore, we introduced the PCA–LDA diagnostic algorithm analysis to distinguish and analyze the SERS spectra of the secreted proteins in different groups.

#### 3.5.1. CNE1

**Figure 7:**
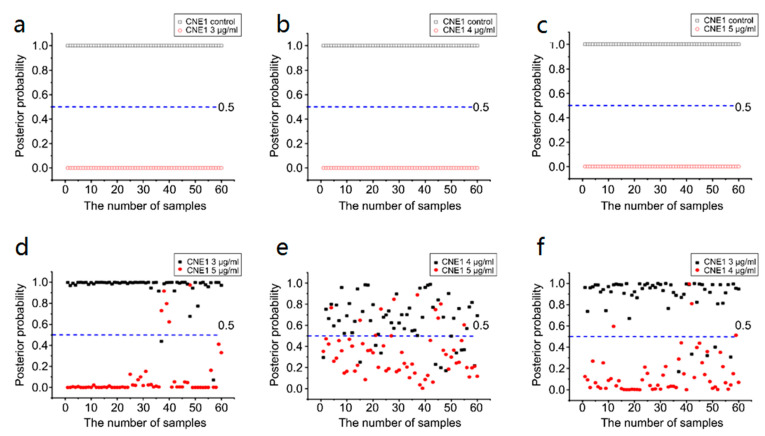
Scatter plots of the posterior probabilities in CNE1 cell-secreted proteins: (**a**) control group versus 3 μg/mL group, (**b**) control group versus 4 μg/mL group, (**c**) control group versus 5 μg/mL group, (**d**) 3 μg/mL group versus 5 μg/mL group, (**e**) 4 μg/mL group versus 5 μg/mL group, and (**f**) 3 μg/mL group versus 4 μg/mL group. The discrimination thresholds in each group are all 0.5.

Figure 7a–c show the posterior probability of the CNE1 cell-secreted proteins analyzed using the PCA–LDA algorithm in the 3, 4, and 5 μg/mL groups (versus the control group). Each group contains 60 samples. The three important diagnostic indices, namely, sensitivity, specificity, and accuracy, were 100% each. These results indicated that SERS could completely distinguish the control group from the experimental group.

Figure 7d–f display the posterior probability between each experimental group. In the 3 μg/mL group versus the 4 μg/mL group, the sensitivity, specificity, and accuracy were 91.7, 93.3, and 92.5%, respectively. In the 3 μg/mL group versus the 5 μg/mL group, these values were 96.7, 91.7, and 94.2%, respectively. Finally, in the 4 μg/mL group versus the 5 μg/mL group, these values were 78.3, 81.7, and 80.0%, respectively.

Figure 7 also shows the optimal discrimination thresholds. The results in the control versus 3 μg/mL groups, control versus 4 μg/mL groups, control versus 5 μg/mL groups, 3 μg/mL versus 4 μg/mL groups, 3 μg/mL versus 5 μg/mL groups, and 4 μg/mL versus 5 μg/mL groups are 0.5 each.

The SERS spectral changes become increasingly obvious with the increase in cisplatin concentration. This result indicates that cisplatin increases the light scattering of secreted proteins, which reflects that conformation of secreted proteins changes after different concentrations of drug treatment.

**Figure 8:**
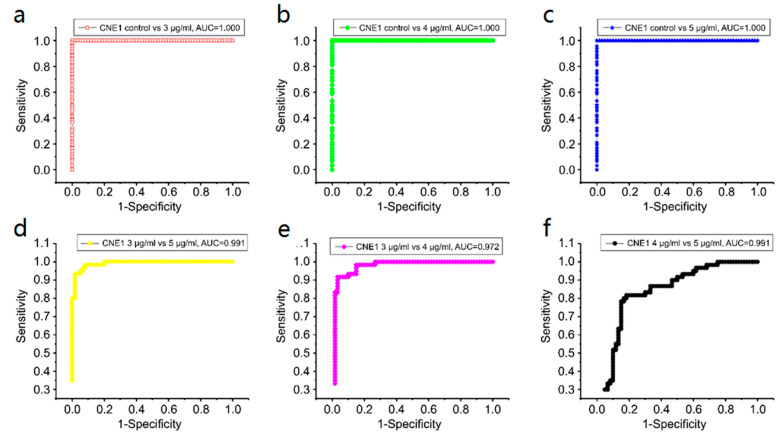
Receiver operating characteristic (ROC) curves of the discrimination results calculated using principal component analysis–linear discriminant analysis in CNE1: (**a**) control group versus 3 μg/mL group, (**b**) control group versus 4 μg/mL group, (**c**) control group versus 5 μg/mL group, (**d**) 3 μg/mL group versus 5 μg/mL group, (**e**) 3 μg/mL group versus 4 μg/mL group, and (**f**) 4 μg/mL group versus 5 μg/mL group.

Receiver operating characteristic (ROC) curves can further verify the capability of the SERS discrimination of secreted proteins (see Figure 8). Table 3 and Table 4 summarize all diagnostic results. The integration areas under the ROC curves are listed in Table 4.

#### 3.5.2. NP69

**Figure 9:**
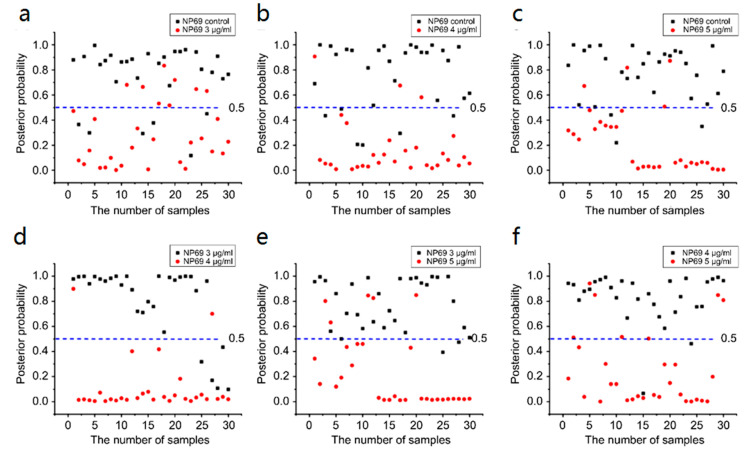
Scatter plots of the posterior probabilities in the NP69 cell-secreted proteins: (**a**) control group versus 3 μg/mL group, (**b**) control group versus 4 μg/mL group, (**c**) control group versus 5 μg/mL group, (**d**) 3 μg/mL group versus 4 μg/mL group, (**e**) 3 μg/mL group versus 5 μg/mL group, and (**f**) 4 μg/mL group versus 5 μg/mL group. The discrimination thresholds are all 0.5.

Figure 9 shows the posterior probability of NP69 cell-secreted proteins analyzed using the PCA–LDA algorithm in each group, which contained 30 samples. Figure 9 also shows the optimal discrimination thresholds. The result in each group is 0.5.

**Figure 10:**
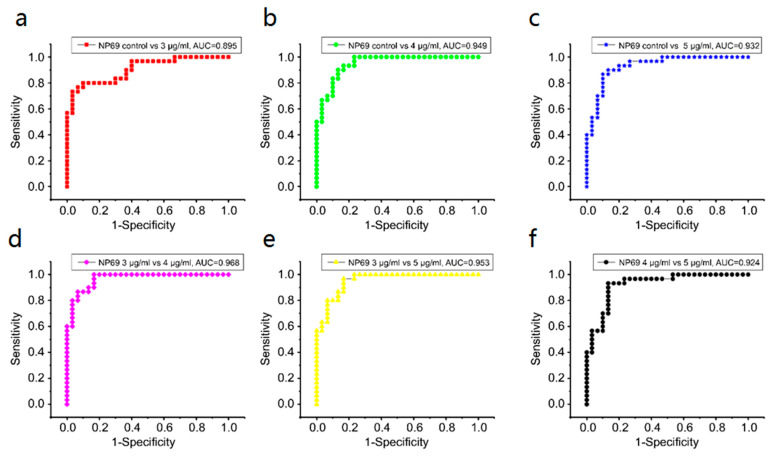
Receiver operating characteristic (ROC) curves of the discrimination results calculated from principal component analysis–linear discriminant analysis in NP69: (**a**) control group versus 3 μg/mL group, (**b**) control group versus 4 μg/mL group, (**c**) control group versus 5 μg/mL group, (**d**) 3 μg/mL group versus 4 μg/mL group, (**e**) 3 μg/mL group versus 5 μg/mL group, and (**f**) 4 μg/mL group versus 5 μg/mL group.

Table 5 and Table 6 summarize all diagnostic results. Table 6 lists the results of sensitivity, specificity, and accuracy among NP69 groups in detail. Figure 9 shows a posterior probability diagram based on PCA–LDA diagnostic model. To further evaluate the performance of secreted protein-SERS in the differential diagnosis of cisplatin response, we generated an ROC curve based on PCA–LDA data, as shown in Figure 10. In Figure 10a, in the control group versus the 3 μg/mL group, the sensitivity, specificity, and accuracy are 79.3, 71.0, and 75.2%, respectively, and the AUC is 0.895. However, in the 3 μg/mL group versus the 5 μg/mL group, the sensitivity, specificity, and accuracy are 86.7, 83.3, and 85.0%, respectively, and the AUC is 0.953.

These results further show that a combination of secreted protein-SERS technology with the PCA–LDA diagnosis algorithm has the potential to become a rapid and noninvasive method for cellular drug response detection and diagnosis.

ROC curves can further verify the capability of the SERS discrimination of secreted proteins (see Figure 10). Table 6 lists the integration areas under the ROC curves. Table 5 and Table 6 summarize all the diagnostic results.

## 4. Discussion

A control experiment was performed before the cell experiment. Specifically, cisplatin at the concentrations of 3, 4, and 5 μg/mL was prepared, and after standing for 24 h, extraction was conducted according to the steps for extracting the cell-secreted proteins. The results showed that no substance was extracted. We believed that cisplatin was sufficiently dissolved and did not affect the SERS spectra of the extracted secreted proteins. Hence, we did not measure the Raman spectra of the silver colloids with cisplatin alone. Meanwhile, we have confirmed that the spectra of silver colloids and cell-secreted proteins did not overlap (see Figure 1b and Figure 4a). We also tried to detect cell-secreted proteins without silver colloids several times; however, because the amount of secreted proteins of NPC is too low, we could not obtain clear images. Therefore, we displayed only figures measured with silver colloids in our study.

The CCK8 assay kit is widely used for in vitro cytotoxicity studies. The results obtained in this study showed that the detection accuracy of the CCK8 kit is not sufficiently high to distinguish between the cytotoxicity of cisplatin with a concentration difference of 1 μg/mL. As an alternative, the SERS spectrum of the cell-secreted protein in combination with PCA–LDA enables high-resolution detection of cisplatin response. If highly differentiated nasopharyngeal carcinoma cell CNE1 is considered as an example, the accuracy of the method for distinguishing the cellular drug response of 3 and 4 μg/mL of cisplatin can reach 91.7%. The accuracy rate for distinguishing between the cellular drug response of 4 and 5 μg/mL of cisplatin can be up to 80%. After the CNE1 cell line is replaced with nasopharyngeal epithelial cell NP69, the accuracy rate of this method for distinguishing between the cellular drug response of 3 and 4 μg/mL of cisplatin can reach 86.8%. The accuracy rate for distinguishing between the cellular drug response of 4 and 5 μg/mL of cisplatin is also 86.8%.

Furthermore, we found that cell-secreted proteins are directly related to and can be used for the analysis of apoptosis. The experimental results showed that the peak intensities of the SERS spectra of the secreted proteins in CNE1 cells at 653 cm^−1^ and 1319 cm^−1^ increased with the increase in drug concentration. Furthermore, the peak intensity of the SERS spectrum of the secreted protein in NP69 cells at 679 cm^−1^ decreased with the increase in drug concentration. The study on small-cell lung cancer in 2019 conducted by Wang et al. from Southwest Medical Center of the University of Texas supported our conclusion [26]. Researchers used high-resolution tandem mass spectrometry to ascertain that IGF-BP5 is a specific secreted protein of ASCL1-high-expression small-cell lung cancer. The expression level of IGF-BP5 was negatively correlated with ASCL1 inhibitor-induced apoptosis.

The band at the 636 cm^−1^ Raman line has been attributed to the stretching vibrations of the C−S bond (L−Tyrosine or lactose) [22] in the CNE1 control group. After treatment with low concentrations of cisplatin, the intensity of the 636 cm^−1^ Raman line decreased and shifted to the 653 cm^−1^ Raman line, which indicated that changes occurred in the conformation of the C−S bond and the content of amino acid and sugars in CNE1 cells. The band at the 653 cm^−1^ Raman line has been attributed to stretching vibrations of C−S bond gauche [24]. The intensity of the 653 cm^−1^ Raman line gradually increased. When the concentration of cisplatin increased by 1 μg/mL, the intensity of the SERS peak of 653 cm^−1^ increased by approximately 20%. However, its position did not change as the concentration of cisplatin increased. These changes may suggested that the increase in gauche conformation in CNE1-secreted proteins. However, the tentative amino acid assignments of 653 cm^−1^ Raman line were not clear. Some studies suggested that the 653 cm^−1^ Raman line has been attributed to alanine [27], methionine [24], cystine [28], or nucleic acid [29].

Similarly, due to low concentrations of cisplatin, the intensity of the 1335 cm^−1^ Raman line also decreased and shifted to the 1319 cm^−1^ Raman line in CNE1 cells. The intensity of the 1319 cm^−1^ Raman line gradually increased as the concentration of cisplatin increased. When the concentration of cisplatin increased by 1 μg/mL, the intensity of the SERS peak of 1319 cm^−1^ increased by 5~16%. The band at the 1319 cm^−1^ and the 1335 cm^−1^ Raman line has been attributed to the CH_3_CH_2_ twisting mode of collagen [30] and the CH_3_CH_2_ wagging mode of collagen [31], respectively. These results suggested that low concentrations of cisplatin induced the change of the CH_3_CH_2_ vibration mode and the increase in collagen content in CNE1-secreted proteins. This phenomenon may be related to the increased levels of mitochondrial-reduced nicotinamide adenine dinucleotide phosphate (NADPH). NADPH is usually used as a reducing agent for biosynthesis. As a hydrogen donor, NADPH is involved in various metabolic reactions in the body, as well as the biotransformation of drugs, toxins, and certain hormones. Another important mechanism of cisplatin cytotoxicity is to increase NADPH in mitochondria [32], produce reactive oxygen species, promote mitochondrial rupture, and subsequently initiate apoptosis [33]. The increase in NADPH activates the proline PRODH/POX pathway, thereby leading to an increase in proline synthesis [34]. Proline is an essential amino acid for collagen synthesis. Therefore, the increased collagen secretion in CNE1 cells may be related to the cisplatin-induced increase in NADPH.

The SERS peak at 679 cm^−1^ has been attributed to glutathione. When the concentration of cisplatin increased from 3 μg/mL to 4 μg/mL, the intensity of the SERS peak of 679 cm^−1^ decreased by approximately 83%. Glutathione, which is ubiquitous in organisms, is an important antioxidant that can bind to a variety of chemicals and their metabolites. It can remove oxygen ions and other free radicals in the body, resist oxidation, and detoxify and maintain the integrity of the erythrocyte membrane. In addition, it has a variety of physiological functions, such as maintaining DNA biosynthesis, normal growth of cells, and cellular immunity. In a study conducted by Cadoni et al., it was shown that glutathione could form complexes with cisplatin, thereby resulting in the loss of cisplatin activity [35]. High expression of glutathione was also observed in some cisplatin-resistant cell lines. Therefore, the decrease in the secreted protein glutathione in CNE1 cells may be related to the formation of “glutathione–cisplatin” complexes.

From Figure 4b, we could easily find that the 1449 cm^−1^ Raman peak in the control group shifted to 1445 cm^−1^, and the intensity was stronger after treatment with cisplatin. Previous studies indicated that the 1449 cm^−1^ peak is assigned to the CH2 and CH3 bending vibrations, and the 1445 cm^−1^ peak is assigned to the CH2 bending vibration. The intense C-H bending vibration is characteristic of these lipid materials. In addition, the SERS peak at 1445 cm^−1^ was reported to be sensitive to the hydrophobicity of the protein environment. Thus, an increase at the 1445 cm^−1^ Raman peak may imply to an increase in the hydrophobicity of cell-secreted proteins induced by cisplatin. Furthermore, the SERS peak at 1668 cm^−1^ in the control group also shifted to 1673 cm^−1^, and the intensity was significantly stronger. Generally, a β-sheet structure was attributable to proteins with an amide I band centered at 1665–1680 cm^−1^. The intensity increases in amide I (1673 cm^−1^) may be correlated with an increase in the β-sheet content. A shift from 735 cm^−1^ to 728 cm^−1^ was also observed. The Raman line in 600–750 cm^−1^ generally indicated the stretching vibrations of the C-S bond of the methionine and cysteinyl residues. The Raman shift depends on the conformation of the C-S bond. The shift from 735 cm^−1^ to 728 cm^−1^ showed that conformation of the C-S bond was changed in cell-secreted proteins after treatment with cisplatin [36,37].

Figure 4a and Figure 5a showed that there are eleven identical Raman peaks for two types of cells. Only three characteristic Raman lines are different for carcinoma cells and epithelial cells (636, 980, and 1090 cm^−1^ for CNE1 cells, whereas 679, 958, and 1072 cm^−1^ for NP69 cells). The SERS peak at 636 cm^−1^ and 679 cm^−1^ is assigned to the stretching vibrations of C-S bond. The shift from 636 cm^−1^ to 679 cm^−1^ showed that conformation changes of the C-S bond in two types of cell-secreted proteins. The SERS peak at 958 cm^−1^ is assigned to ${\mathrm{PO}}_{4}^{3-}$ symmetric stretching vibration, whereas 980 cm^−1^ Raman line is assigned to ${\mathrm{SO}}_{4}^{2-}$ symmetric stretching vibration. The intensity of 980 cm^−1^ Raman line in CNE1-secreted proteins was stronger than that in NP69-secreted proteins, which implied enhancement of ${\mathrm{SO}}_{4}^{2-}$ symmetric stretching vibration in CNE1-secreted proteins. The SERS peak at 1090 cm^−1^ is assigned to the triple degenerate v_3_ asymmetric P-O stretching mode, whereas at 1072 cm^−1^, the Raman line may assign to the v_1_ vibrational mode of carbonate (CO_3_^2−^) intercalation in the apatite complex. For Raman spectrum, the intensity of 1090 cm^−1^ Raman line in CNE1-secreted proteins was stronger than that in NP69-secreted proteins, which implied cleavage of the P-O bond in NP69-secreted proteins. Furthermore, the three characteristic Raman lines could be used to distinguish between CNE1 cells and NP69 cells [38,39].

We introduced the PCA–LDA statistical method to identify and discriminate the SERS spectra of secreted proteins. Figure 7a–f and Figure 9a–f show that most of the clusters of secreted protein samples from the control group and cisplatin-treated groups have been broadly separated into two parts except for the overlap of a few samples. In CNE1, the trend of separation between different secreted protein clusters is more apparent under the concentrations of 3 and 5 μg/mL. In NP69, the trend of separation between different secreted protein clusters is more apparent under the concentrations of 3 and 4 μg/mL. With the increase in the concentration of cisplatin, the cluster points of secreted protein samples from the control group and different concentrations of the cisplatin-treated group gather to their respective ends, indicating that the variance between different secreted protein groups is larger; meanwhile, the variance within the same groups is smaller. These results indicated that secreted protein SERS technology combined with PCA–LDA could be a sensitive and accurate tool to study the cellular response to low-concentration cisplatin treatments of nasopharyngeal cells.

In 2020, Du et al. [40] developed a novel recyclable SERS-based immunoassay, which has promising prospects in the applications of clinical measurements for cancer diagnosis. In the future, we propose further analyzing the molecular weight and species of the extracted secreted proteins using SERS-based immune technology.

In summary, this experiment showed that slight changes in secreted proteins related to cellular drug response could be sensitively detected by the optical characteristics of SERS. Hence, the secreted protein-SERS technique is promising for evaluating the therapeutic cellular drug response and the effect of chemotherapeutic medicines.

## 5. Conclusions

The cellular drug response assay based on the SERS spectrum of the secreted proteins can provide a higher detection accuracy than those of conventional assay techniques, such as CCK8. The results revealed that the cisplatin response at a concentration difference of 1 μg/mL could be detected sufficiently, and the diagnostic sensitivity and specificity were high. This exploratory study shows that although the cell activity has not changed, obvious differences of amino acid metabolism in CNE1 cells have emerged. Amino acid metabolism is closely associated with cell proliferation, differentiation, immune response, and drug resistance. Our findings provided some new insights into the mechanism of low-dose chemotherapy and a new strategy to search for effective anti-tumor targets for clinicians.

## Figures and Tables

**Table 1:** Information regarding the top eight most abundant secreted proteins in CNE1 and NP69 in different groups.

Sample	No.	Accession	Gene	Mw(kD)	iBAQ [%]
CNE1 control	1	P06733	ENO1	47.169	2.813166208
	2	P23528	CFL1	18.502	1.69781084
	3	P07195	LDHB	36.638	1.504842291
	4	P04406	GAPDH	36.053	1.420498423
	5	P14618	PKM	57.937	1.234203547
	6	F8W6I7	HNRNPA1	33.155	1.140956715
	7	P07737	PFN1	15.054	1.105501051
	8	P18669	PGAM1	28.804	1.101198662
CNE1 3 µg/mL	1	P62805	H4C1	11.367	2.221588586
	2	P07737	PFN1	15.054	1.892088776
	3	P06733	ENO1	47.169	1.60544963
	4	P23528	CFL1	18.502	1.411833
	5	P07900	HSP90AA1	84.66	1.387090092
	6	P00338	LDHA	36.689	1.22050137
	7	P68104	EEF1A1	50.141	1.078141768
	8	P63261	ACTG1	41.793	1.063363627
CNE1 4 µg/mL	1	P62805	H4C1	11.367	2.105799202
	2	P07737	PFN1	15.054	1.664594022
	3	P06733	ENO1	47.169	1.607366138
	4	P23528	CFL1	18.502	1.42540016
	5	P07900	HSP90AA1	84.66	1.404212341
	6	P00338	LDHA	36.689	1.227854903
	7	P62937	PPIA	18.012	1.033113917
	8	P07195	LDHB	36.638	1.026684867
CNE1 5 µg/mL	1	P62805	H4C1	11.367	2.09864077
	2	P06733	ENO1	47.169	1.731257867
	3	P07737	PFN1	15.054	1.622628465
	4	P23528	CFL1	18.502	1.555246031
	5	P07900	HSP90AA1	84.66	1.390382025
	6	P00338	LDHA	36.689	1.261067207
	7	P07195	LDHB	36.638	1.077796895
	8	P62937	PPIA	18.012	1.070724217
NP69 control	1	P06733	ENO1	47.169	1.355017617
	2	P10599	TXN	11.738	1.293302386
	3	P23528	CFL1	18.502	1.234233114
	4	P62805	H4C1	11.367	1.223220199
	5	P63261	ACTG1	41.793	1.217642229
	6	F8W6I7	HNRNPA1	33.155	1.199192021
	7	P04406	GAPDH	36.053	1.182100549
	8	P04083	ANXA1	38.714	1.070469638
NP69 3 µg/mL	1	P06733	ENO1	47.169	1.9117112
	2	P63261	ACTG1	41.793	1.666133678
	3	P68104	EEF1A1	50.141	1.536606509
	4	P07355	ANXA2	38.604	1.417935664
	5	P62805	H4C1	11.367	1.350426802
	6	P04083	ANXA1	38.714	1.290779416
	7	P14618	PKM	57.937	1.268941984
	8	P04406	GAPDH	36.053	1.228424188
NP69 4 µg/mL	1	P06733	ENO1	47.169	3.059654209
	2	P04406	GAPDH	36.053	1.937876617
	3	P63261	ACTG1	41.793	1.690142515
	4	P07737	PFN1	15.054	1.504906682
	5	P0DMV9	HSPA1B	70.052	1.365441956
	6	P63241	EIF5A	16.832	1.317286377
	7	P60174	TPI1	30.791	1.317071236
	8	F8W6I7	HNRNPA1	33.155	1.308519408
NP69 5 µg/mL	1	P06733	ENO1	47.169	12.91194799
	2	P63261	ACTG1	41.793	2.849968877
	3	P07355	ANXA2	38.604	1.64815391
	4	P68104	EEF1A1	50.141	1.575224042
	5	P07737	PFN1	15.054	1.411233695
	6	P00338	LDHA	36.689	1.370609586
	7	P0DMV9	HSPA1B	70.052	1.275186217
	8	P23528	CFL1	18.502	1.20724736

**Table 2:** Surface-enhanced Raman scattering peak position and tentative assignments [22,23,24,25].

Peak Position (cm^−1^)	Tentative Assignments
562,563	Proline
636	L-Tyrosine, lactose
653	C–S bond
679	Glutathione
730–735	Phosphatidylserine
888	Protein bands; structural protein modes of tumors
955–958	Protein
980	Protein
1001–1004	Phenylalanine, C–C skeletal
1072	Collagen
1090	Symmetric phosphate stretching vibrations
1267	Amide III (collagen assignment)
1319–1321	Collagen assignment; Amide III
1335	Collagen (protein assignment)
1448–1449	Collagen (protein assignment)
1668	Structural protein modes of tumors

**Table 3:** Statistical results of different groups in CNE1.

Peak Position (cm^−1^)	Control vs. 3 μg/mL	Control vs. 4 μg/mL	Control vs. 5 μg/mL	3 μg/mL vs. 4 μg/mL	3 μg/mL vs. 5 μg/mL	4 μg/mL vs. 5 μg/mL
653	<0.001	<0.001	<0.001	<0.001	<0.001	0.037
1319	<0.001	<0.001	<0.001	<0.001	<0.001	0.055

**Table 4:** Diagnostic results of CNE1 cell-secreted proteins using principal component analysis and linear discriminant analysis (PCA–LDA) approach.

Diagnostic Combinations	Predicted Results			
	Sensitivity (%)	Specificity (%)	Accuracy (%)	AUC
Control vs. 3 μg/mL	100	100	100	1.000
Control vs. 4 μg/mL	100	100	100	1.000
Control vs. 5 μg/mL	100	100	100	1.000
3 μg/mL vs. 4 μg/mL	91.7	93.3	92.5	0.972
3 μg/mL vs. 5 μg/mL	96.7	91.7	94.2	0.991
4 μg/mL vs. 5 μg/mL	78.3	81.7	80.0	0.838

**Table 5:** Statistical results of different groups in NP69.

Peak Position (cm^−1^)	Control vs. 3 μg/mL	Control vs. 4 μg/mL	Control vs. 5 μg/mL	3 μg/mL vs. 4 μg/mL	3 μg/mL vs. 5 μg/mL	4 μg/mL vs. 5 μg/mL
679	<0.001	<0.001	<0.001	<0.001	<0.001	0.711

**Table 6:** Diagnostic results of NP69 cell-secreted proteins using principal component analysis and linear discriminant analysis (PCA–LDA) approach.

Diagnostic Combinations	Predicted Results			
	Sensitivity (%)	Specificity (%)	Accuracy (%)	AUC
Control vs. 3 μg/mL	79.3	71.0	75.2	0.895
Control vs. 4 μg/mL	76.7	90.0	83.4	0.949
Control vs. 5 μg/mL	80.0	80.0	80.0	0.932
3 μg/mL vs. 4 μg/mL	80.0	93.3	86.7	0.968
3 μg/mL vs. 5 μg/mL	86.7	83.3	85.0	0.953
4 μg/mL vs. 5 μg/mL	93.3	76.7	85.0	0.924

## Data Availability

The raw/processed data required to reproduce these findings cannot be shared at this time, as the data forms a part of an ongoing study.
